# Occurrence of *Brettanomyces bruxellensis* on Grape Berries and in Related Winemaking Cellar

**DOI:** 10.3389/fmicb.2019.00415

**Published:** 2019-03-07

**Authors:** Lucia Oro, Laura Canonico, Valentina Marinelli, Maurizio Ciani, Francesca Comitini

**Affiliations:** Department of Life and Environmental Sciences, Marche Polytechnic University, Ancona, Italy

**Keywords:** *Brettanomyces bruxellensis*, grape berry, winery, molecular characterization, ecological distribution

## Abstract

The spoilage yeasts belonging to the genus *Dekkera* (anamorph *Brettanomyces*) are associated with the fermentation process and can be responsible for off-flavors in wine. *Brettanomyces bruxellensis* is difficult to isolate from natural environments because of its low diffusion, low presence on the grape surface and low competition capacity, slow growth, and VBNC (viable but not culturable) state, even when selective media are used. In this study, to investigate the origins and occurrence of *B. bruxellensis* in winemaking, a total of 62 samples from grapes, winery environment, and fermenting musts were taken through direct isolation with a selective medium. *B. bruxellensis* was not directly detected in the grape samples but was instead widely isolated from the winery environment samples. However, using a combination of enrichment and selective media, eight of fifteen grape samples were positive for *B. bruxellensis.* Analysis of the genetic traits of the isolates indicated a strict relationship among the strains from the vineyard and the winery. Isolates from the vineyard and the winery were both part of the more common and dominant biotypes suggesting that the vineyard may be the contamination source of *B. bruxellensis* in the winery environment. For this, grapes may represent the possible primary origin source from which a flow toward the winery environment originates. On the other hand, the wide occurrence of *B. bruxellensis* in winery indicates that this environment can be considered as the favorable ecological niche for colonization and diffusion of these yeast.

## Introduction

*Brettanomyces bruxellensis* is a yeast that has been isolated from several natural ecological niches, and it is intrinsically linked with various industrial fermented products, such as wine, beer, cider, kombucha tea, and bioethanol (Martens et al., 1997; Greenwalt et al., 2000; Morrissey et al., 2004; Silva et al., 2004; Teoh et al., 2004; Smith and Divol, 2016). The role of *B. bruxellensis* in these fermentation processes is often ambiguous. In some industrial processes, as beer production, the presence of *B. bruxellensis* can be considered favorable, as is produces aromas contributing to the specific style of some specialty beers (Gilliland, 1961; Spitaels et al., 2014). However, the same aroma compounds are considered as off-flavors in the production of other beer styles and of wines (Verachtert, 1992; Wedral et al., 2010; Steensels et al., 2015). In winemaking, the main factor that affects the sensory properties that are imparted by *B. bruxellensis* is the production of 4-ethylphenol, 4-ethylguaiacol, and tetrahydropiridine, which are considered unpleasant aromas and are defined as the “Brett character” (Heresztyn, 1986; Chatonnet et al., 1992; Dias et al., 2003; Ciani and Comitini, 2014; Valdetara et al., 2017).

*Brettanomyces bruxellensis* is mainly associated with wine aging in barrels, and with stuck or sluggish fermentation (Suárez et al., 2007). This is possible due to the physiological traits of *B. bruxellensis*, as this yeast shows high ethanol tolerance, SO_2_ resistance and growth at low nutrient availability (Loureiro and Malfeito-Ferreira, 2003; Barata et al., 2008; Coulon et al., 2011; Steensels et al., 2015; Avramova et al., 2018). Compared with white wines, red wines are particularly susceptible to *B. bruxellensis* contamination due to their higher pH, lower SO_2_ addition, higher polyphenols content, and barrel aging (i.e., oxygen availability) (Benito et al., 2009). Due to this major role for *Dekkera/Brettanomyces* yeast in industrial fermentation processes, their key phenotypic characteristics have been extensively studied, and especially for *B. bruxellensis*. Although many of these *Dekkera/Brettanomyces* strains occupy similar ecological niches to *Saccharomyces cerevisiae*, their general physiology and phenotypic traits show remarkable differences (Nardi et al., 2010; Steensels et al., 2015).

Since the 1980’s, numerous studies have speculated on the occurrence and diffusion of *Brettanomyces* spp. in winemaking (Pretorius, 2000; Suárez et al., 2007; Agnolucci et al., 2009; Campolongo et al., 2010; Di Toro et al., 2015), although their origin is not well defined yet (Aguilar-Uscanga et al., 2000; Curtin and Pretorius, 2014; Avramova et al., 2018). Many studies have associated this spoilage yeast with poor hygienic practices in the cellar, presence of cellobiose and micro-oxygenation related to the use of wooden barrel, together with high ethanol content (Chatonnet et al., 1999; Renouf and Lonvaud-Funel, 2007; Boulton et al., 2013). It was only in 2007 that the presence of *B. bruxellensis* on the grape berry surface was clearly demonstrated, using an enrichment medium (Renouf and Lonvaud-Funel, 2007). The use of this formulated medium represents a useful tool to detect *B. bruxellensis*, as this yeast appears to have low presence and to lack competitive ability in ecological niches such as the grape berry surface. Moreover, *B. bruxellensis* could entry in VBNC state (Capozzi et al., 2016). Even using more sensitive recent techniques as transcriptome approach/new generation sequencing (NGS) *B. bruxellensis* was not isolated (Bokulich et al., 2014; Pinto et al., 2014; Kecskeméti et al., 2016; Morgan et al., 2017). Albertin et al. (2014) compared *B. bruxellensis* strains detected from grape and wine by microsatellite analysis. This study shows that there is not strict genetic pattern depending on the substrate. Namely, strains isolated from grape clustered together with strains coming from wine, suggesting a strong connection between grapes and the cellar.

The present study investigated the occurrence of *B. bruxellensis*. The presence of this yeast was monitored on the grape berry surface and in the winery during the winemaking process, with the isolated strains characterized by molecular typing.

## Materials and Methods

### Sampling

The 12 sites selected for this *B. bruxellensis* isolation and characterization study are part of a farm of over 100 ha of vineyards situated in the hills to the south-west of Florence, near the town of Vicarello, at 50 km from the winery in Montespertoli (FI), thus avoiding possible cross-contamination (Goddard et al., 2010). Indeed, the farm is only held the cultivation and harvesting grapes. The grapes mechanical harvesting, were loaded into wagons and transported to the cellar.

In this situation any anthropogenic contamination from the farm to the winery and vice versa was excluded, since there were different workers involved in the winery activity and in the vineyard management.

The sampling campaign was performed during the 2014 harvest, as grape samples collected from 12 locations in the vineyard that contained four different grapevine varieties: “Syrah,” “Merlot,” “Cabernet Franc,” and “Cabernet Sauvignon.” Each vineyard sampling site had very similar characteristics in terms of age, pruning system, canopy management, and sun exposure, and they were also characterized by similar climatic conditions (September: mean air temperature, 28°C; rainfall, 70.5 mm).

The vineyard sampling sites were almost 800 m from each other, and they were randomly selected, although taking into account the position of the grapes harvested (i.e., beginning, middle, and end of grapevines rows). A total of 26 different grape samples of 2–3 kg each were analyzed. Samples were also collected from the winery environment: from the transport trailer (100 mL of grape juice from the bottom of the grape bin), the destemmer (1 Kg of grapes), the washing water of the valves between the destemmers and the tanks (50 mL of residue liquid), the air (using an SAS-Super 180 high microbiological air sampler; Bioscience International, Rockville, MD, United States). During the fermentation seven vats of 450 hL were monitored at the start, middle, and end of the fermentation process (one Syrah, two Merlot, two Cabernet Franc, and two Cabernet Sauvignon).

All of the samples were collected aseptically and stored at 4°C until arrival in the laboratory (within 6 h of collection), and then they were processed immediately.

### Detection and Isolation of *B. bruxellensis* From Grapes and the Winery Environment

The refrigerated bags that contained the harvested grape berries were opened in the laboratory under sterile conditions, and 45 berries per sample were taken and placed into a 250 mL sterile flask containing 150 mL of the enrichment *B. bruxellensis* (EBB) medium (Renouf and Lonvaud-Funel, 2007). The EBB medium had the following composition: 200 mL/L red grape juice (commercial grape juice; Folicello, Modena, Italia with 19.1% sugar, nitrogen content YAN 115 mgN/L and pH 3.4), 40 mL/L ethanol (VWR International, Milan, Italy), 1.5 g/L malt extract (Sigma-Aldrich, Milan, Italy), 1.5 g/L yeast extract, 0.5 g/L (NH_4_)_2_SO_4_ (Sigma-Aldrich, Milan, Italy), 0.2 g/L MgSO_4_, and 0.5 mL/L Tween 80 (Merck, Hohenbrunn, Germany). The pH was adjusted to 5.0 with sodium hydroxide. The EBB medium was also supplemented with 0.2 g/L (w/v) biphenyl (Fluka, Steinheim, Switzerland) and 0.05 g/L (w/v) chloramphenicol (Sigma-Aldrich, Milan, Italy), to limit mold and bacteria development, respectively. The prepared flasks were incubated at 25°C with gentle agitation (120 rpm), and after 2 h the first sample was taken for analysis on Petri dishes with the *Dekke*ra/*Brettanomyces* differential medium (DBDM) (Rodrigues et al., 2001). Subsequently, to monitor the evolution of the *B. bruxellensis* population, further sampling of the EBB medium cultures was carried out on days 10, 40, and 80. These determinations of viable and culturable cells of *Dekkera/Brettanomyces* were carried out semi quantitatively, as absent (-) or present (+). To detect *Brettanomyces* yeasts in the winery, microbial sampling (1 mL) was performed at the following steps: (i) from the trailer, before load discharge into the destemmer; (ii) during the pressing and destemming of the grapes; (iii) during grape juice fermentation (at the start, middle, and end of process). The following grape varieties were monitored: “Syrah,” “Merlot,” “Cabernet Franc,” and “Cabernet Sauvignon.”

Fermentation was carried out in stainless steel fermentation tanks (450 hL) at 22 to 27°C. All fermentations were inoculated with *S. cerevisiae* commercial starter strain (Zymaflore F15 – Laffort). Samples for *B. bruxellensis* analysis were also taken from the connecting fermenter valves, as 10 mL of the residual washing water, with 1 mL from each solution used on Petri dishes with the DBDM medium. Finally, analysis of the air was performed using a microbiological air sampler that can suck in multiple aliquots of air (according to need), and project it directly onto a Petri dish with DBDM medium. This test was performed at the beginning and in the middle of the harvesting campaign. Three samples were also taken from sites near the exhaust (in the winery square) and near the destemmer, at the start and in the middle of the harvesting.

### Molecular Identification and Biotyping of *B. bruxellensis*

Preliminary molecular identification of 250 presumptive *B. bruxellensis* strains was carried out to confirm the genus and species of these yeast. For this, all of the isolated strains underwent DNA extraction (Stringini et al., 2009) and sequence analysis of the 26S-D1/D2 region of rDNA, which identified the specific nucleotide sequences of the individual species (Kurtzman and Robnett, 1998).

The DNA extracted from the *B. bruxellensis* strains underwent biotyping using the RAPD-PCR primers M13 (5′-GAG GGT GGCGGT TCT-3′); M14 (5′-GAGGGT GGG GCC GTT-3′); OPC20 (5′-ACTTCGCCAC-3′); OPK03 (5′-CCAGCTTAGG-3′) and the minisatellite primers PIR1 (forward: 5′-GCCACTACTGCTTCCTCCAA-3′; reverse: 5′-TGGACCAACCAGCAGCATAG-3′) and PIR3 (forward: 5′-TCCTCCGTCGCCTCATCTAA-3′; reverse: 5′-GGCACTGAGAACCAATGTGC-3′). For molecular typing the type strain coming from University of Perugia collection (DBVPG 6706) was included. The conditions used for the amplification protocols were as described by Crauwels et al. (2014) and Canonico et al. (2015).

### Statistical Analysis

The genotypic analysis of the isolates into biotype clusters was performed using JMP version 11 (JMP SAS-Genomics, United States). The cluster analysis was carried out according to the Ward’s minimum variance method, and it is represented as a constellation plot. This diagram, arranges the samples as endpoints and each cluster join as a new point. The lines represent membership in a cluster. The length of a line between cluster joins approximates the distances between the cluster that were joined.

## Results

### Occurrence of *B. bruxellensis* on Grape Surfaces and in the Winery

At harvest (day 0), all of the grape samples showed a quantitative yeast population of about 10^4^ CFU/mL, without any significant variations among these, with exception of a sample of the “Syrah” variety (code: gS2), with a yeast population two orders of magnitude greater. The main representative yeast belonged to the *Hanseniaspora* and *Pichia* genera, and grew on Wallerstein laboratory (WL) agar medium (data not shown). However, no *B. bruxellensis* strains were detected, even using the EBB medium. After 10 days of incubation of the grapes in EBB medium, *B. bruxellensis* was isolated from only one Syrah sample (code: gS4), while after 40 days, a “Cabernet Sauvignon” variety was positive for *Brettanomyces* strains in two samples (codes: gCS2, gCS5) (Table 1). The last sampling was carried out on day 80, with *B. bruxellensis* isolated from eight different grape samples (Table 1). None of the “Merlot” variety grape samples showed any *B. bruxellensis* throughout these sampling periods. After the further cultivation in DBDM medium, three or four colonies per sample were collected, and the eight positive samples yielded 15 *B. bruxellensis* isolates, which were identified and stored for further molecular characterization.

**Table 1:** Summary of the different grape samples.

Variety	Code	Direct isolation as viable cell counts (CFU × 10^3^berry^-1^)^a^	Detection according to time of isolation (days) after enrichment in EBB, and strain code		
			
			T10	T40	T80
Syrah	gS1	9.6 ± 1.3	–	–	–
	gS2	4600 ± 2500	–	–	–
	gS3	130 ± 140	–	–	gS3a, gS3b
	gS4	300 ± 330	gS4a	gS4b	gS4c
	gS5	16 ± 5	–	–	–
	gS6	13 ± 19	–	–	–
Merlot	gM1	9.6 ± 5.4	–	–	–
	gM2	6.3 ± 1.6	–	–	–
	gM3	2.3 ± 4.7	–	–	–
	gM4	96 ± 33	–	–	–
	gM5	170 ± 90	–	–	–
	gM6	20 ± 5	–	–	–
Cabernet	gCF1	12 ± 11	–	–	gCF1a, gCF1b
Franc
	gCF2	170 ± 80	–	–	gCF2a
	gCF3	200 ± 150	–	–	–
	gCF4	29 ± 62	–	–	–
	gCF5	5.3 ± 2.2	–	–	–
	gCF6	28 ± 4	–	–	–
Cabernet	gCS1	27 ± 30	–	–	gCS1a
Sauvignon
	gCS2	17 ± 55	-	gCS2a	gCS2b
	gCS3	3.9 ± 1.1	–	–	–
	gCS4	14 ± 6	–	–	–
	gCS5	1.6 ± 4.0	–	gCS5a	gCS5b
	gCS6	360 ± 130	–	–	gCS6a, gCS6b
	gCS7	150 ± 220	–	–	–
	gCS8	170 ± 400	–	–	–


*^a^obtained as epiphytic population from 45 berries in 150 mL of EBB per sample; means ± SD.*

In the samples collected from the winery environment, *B. bruxellensis* was not detected from the trailers, the destemming process, or the air samples. The only positive samples were collected from two of the three valves (order of magnitude, 10^4^ CFU/mL) used as connections for the transfer of the grape must from one vat to another. During the fermentation conducted under maceration conditions, only one must sample (code: wF1) was positive for *B. bruxellensis* (order of magnitude, 10^2^ CFU/mL) at the initial fermentation stage, while there was wide occurrence of this *B. bruxellensis* spoilage yeast at the middle (two positive samples out of seven) and the end (four positive samples out of seven) of the process, where ∼10^5^ CFU/mL was achieved (Table 2). Therefore, *B. bruxellensis* was isolated from four of the seven tanks monitored. Following these winery sampling procedures, a total of 24 *B. bruxellensis* strains were isolated, identified and stored for further molecular characterization.

**Table 2:** Summary of the different winery samples.

Sample	Grape variety	Code	Direct isolation of *Brettanomyces bruxellensis*		
			
			Rich medium	Selective medium	
				
			Total viable cell counts (CFU × 10^3^ mL^-1^)^a^	(CFU × 10^3^ mL^-1^)^a^	Code
Trailer	Syrah	wW1	1.9 ± 1.5	–	–
	Merlot	wW2	0.07 ± 0.003	–	–
	Cabernet Franc	wW3	0.46 ± 0.24	–	–
	Cabernet Sauvignon	wW4	4.1 ± 2.4	–	–
Destemmer	Syrah	wD1	1.3 ± 0.9	–	–
	Merlot	wD2	0.079 ± 0.042	–	–
	Cabernet Franc	wD3	0.53 ± 0.36	–	–
	Cabernet Sauvignon	wD4	11 ± 10	–	–
	Cabernet Sauvignon	wD5	3.7 ± 4.2	–	–
Start of fermentation ^b^	Syrah	wF1	1.9 ± 0.9	0.1 ± 0.005	wF1a; wF1b; wF1c
	Merlot	wF2	1.0 ± 1.7	–	–
	Cabernet Franc	wF3	25 ± 22	–	–
	Cabernet Sauvignon	wF4	8.0 ± 1.3	–	–
Middle of fermentation ^b^	Syrah	wF5	190 ± 300	41 ± 0.8	wF5a; wF5b;wF5c
	Merlot	wF6	19 ± 12	–	–
	Cabernet Franc	wF7	230 ± 500	84 ± 1.2	wF7a; wF7b; wF7c
	Cabernet Sauvignon	wF8	4.7 ± 1.3	–	
End of fermentation ^b^	Syrah	wF9	8000 ± 1900	200 ± 5	wF9a; wF9b; wF9c
	Merlot	wF10	19000 ± 2000	242 ± 6	wF10a; wF10b; wF10c
	Cabernet Franc	wF11	25000 ± 0	82 ± 0.6	wF11a; wF11b; wF11c; wF11d
	Cabernet Sauvignon	wF12	9700 ± 3500	100 ± 10	wF12a; wF12b; wF12c
Valves	–	wV1	41 ± 5	32 ± 0	wV1a
	–	wV2	26 ± 14	25 ± 0.9	wV2a
	–	wV3	20 ± 41	18 ± 1	wV3a
Air	–	wA1	0.11 ± 0.01	–	–
	–	wA2	0.23 ± 0.43	–	–
	–	wA3	0.18 ± 0.52	–	–


*^a^Means ± SD.*

*^b^Three vats from Melot, Cabernet Franc, Cabernet Sauvignon must (nine samples for start, middle, and end of fermentation) were negative for *B. bruxellensis*.*

### *B. bruxellensis* From the Grapes and the Winery: Genetic Relationships

**FIGURE 1:**
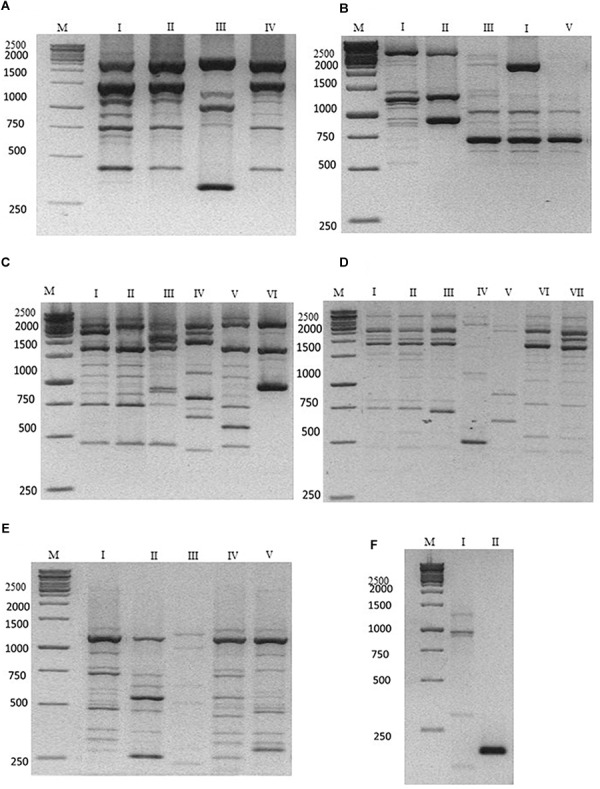
Representative electrophoresis analysis after PCR amplification using **(A)** M14; **(B)** M13; **(C)** OPC20; **(D)** OPK03 RAPD primers; **(E)** PIR1; and **(F)** PIR3 minisatellite primers. Lane M: Gene Ruler 1kb (Fermentas) as indicated on the left of each gel. Lane I–VII **(A–F)** indicate the representative biotypes, as extensively reported in Table 3.

The characterization at strain level carried out using the M13, M14, OPC20 and OPK03 RAPD and the PIR1 and PIR3 minisatellite primers was reported in Figure 1. The use of the OPK03 primer showed the higher discrimination power at strain level with resulting electrophoretic profiles grouped in seven different biotypes (Figure 1D). OPC20 primer grouped the isolated into six biotypes, while the other two RAPD primers, M14 and M13 divided the strains into four and five biotypes, respectively.

The discriminatory power was compared with other PCR-fingerprinting based on minisatellites. In particular, PIR1 and PIR3 showed five and two different electrophoretic profiles, respectively. These last results highlighted a lower discrimination power of PIR3 primer in comparison with RAPD primers. The data obtained are summarized according to the biotype profiles in Table 3.

**Table 3:** The discriminatory power of primers on the *B. bruxellenisis* isolates (see Figure 1).

Primer	*B. bruxellensis* stains according to biotype profiling						
	
	I	II	III	IV	V	VI	VII
M14	gCF2a	wF11a, gCS5a, gCS2a, gCS2b, wF1b, gCS5b, wF5c, gCS6b, gCF1a, gCF1b, gCS1a, wV3a, gS4a, gS4b, gS4c, wF9c, gS3a, gS3b, gCS6a, wF5a, wF7b, wF9b, wF10b, wF10c, wF12c, wV1a, wV2a, wF11b, wF11c, wF12a, wF7a, type strain, wF12b, wF10a, wF5b, wF11d	wF9a	wF7c			
M13	wF11b	wF9a	gCS2a, wF5c, gCS6b, gCS6a, wF5a	gCF2a, gCS2b, wF1b, gCF1a, gCF1b, gCS1a, wV3a, gS4a, gS4b, gS4c, wF9c, gS3a, gS3b, wF7b, wF9b, wF10b, wF10c, wF12c, wV1a, wV2a, wF11a, wF11d, wF11c, wF12a, wF7a, wF7c, wF12b, wF10a, wF5b	type strain, gCS5b, gCS5a		
OPC20	gCS5a, gCS5b, wF5c, gCS6b, gCF1b, wV3a, gS4b, wF9c, gCF1a, gS3a, gS3b, wF10b, wF10c, wF12c, wF11d, wF5b, wF7c, wF11a, gCF2a	gCS2a, gCS2b, wF1b, gCS1a, gS4a, gS4c, gCS6a, wF5a, wF7b, wF9b, wV1a, wV2a, wF11c, wF12a, wF12b, wF10a	type strain	wF11b	wF7a	wF9a	
OPK03	wF10a	wF5b, type strain, wF7a, wF10b	wF12b, wF11c, wF12c, wF7c	wF11b	wF9a	gCS5a, gCS2a, gCS2b, wF1b, gCS5b, wF5c, gCS6b, gCF1b, gCS1a, wV3a, gS4b, wF9c, gCF1a, gS3b, wF5a, wF9b, wF10c, wF12a, wF11a, gCF2a	gS4a, gS4c, gS3a, gCS6a, wF7b, wV1a, wV2a, wF11d
PIR1	wF11d, gCS2a, wF5c, gCS6b, gCS6a, wF5a, gCF2a, gCS2b, wF1b, gCF1a, gCF1b, gCS1a, wV3a, gS4a, gS4b, gS4c, wF9c, gS3a, gS3b, wF7b, wF9b, wF10b, wF10c, wF12c, wV1a, wV2a, wF11a, wF11b, wF11c, wF12a, wF12b, wF5b, type strain, gCS5b, gCS5a	wF10a	wF9a	wF7a	wF7c		
PIR3	gCS2a, wF5c, gCS6b, gCS6a, wF5a, gCF2a, gCS2b, wF1b, gCF1a, gCF1b, gCS1a, wV3a, gS4a, gS4b, gS4c, wF9c, gS3a, gS3b, wF7b, wF9b, wF10b, wF10c, wF12c, wV1a, wV2a, wF11a, wF11c, wF12a, wF12b, wF5b, type strain, gCS5b, gCS5a, wF10a, wF9a, wF7a, wF7c	wF11d, wF11b					


**FIGURE 2:**
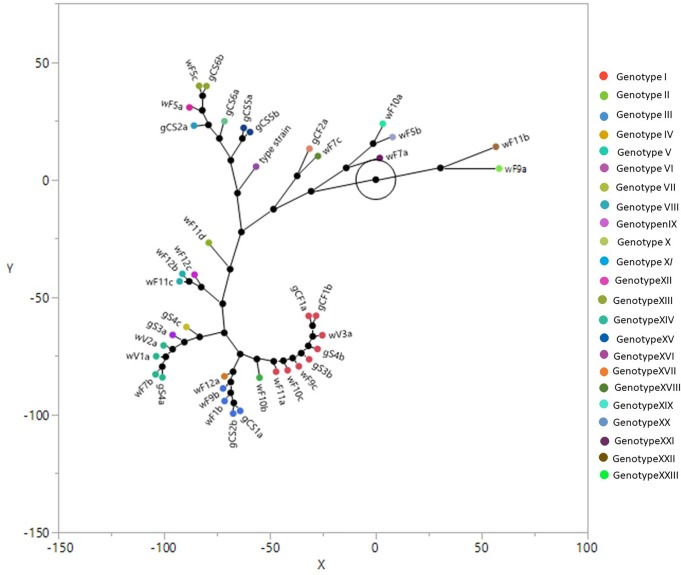
Ward clustering and constellation plot of the *Brettanomyces bruxellensis* biotypes using M13, M14, OPC20 and OPK03 RAPD primers and the PIR1 and PIR3 minisatellite primers. For yeast strain codes, see Tables 1, 2.

The overall typing using the combination of above mentioned primers showed 22 different biotypes as reported in the constellation plot using Ward clustering (Figure 2) and in tree with Bootstrap analysis (Figure 1 and Supplementary Material). The plot showed three main biotypes, containing isolates coming both from grapes and winery. One of these biotypes contained the isolates gCF1a and gCF1b (grape from Cabernet Franc); gS4b and gS3b (grape from Syrah); wF9c, wF10c and wF11c (end of fermentation, winery); wV3a (from valve, winery). This result showed that the isolates coming from winery and grapes are grouped together and could be considered clones of the same genotype. The second largest biotype includes two isolates gCS1a and gCS2b (grape from Cabernet Sauvignon) and three isolates wF11b, wF9b, wF12a (end fermentation, from winery). The last largest biotype is formed by isolates coming from winery environment (middle fermentation and valve) (wF7b, wV1a, and wV2a), and one isolate from Syrah grape (gS4a). The Figure 2 showed also three small biotypes: one includes two isolates coming from two different winery samples at the end fermentation (wF11c, wF12b); the second one is composed by wF5c, isolate coming from winery sample at middle fermentation and gCS6b (grape from Cabernet Sauvignon). The last one contains two strains from the Cabernet Sauvignon (gCS5a and gCS5b). Finally, 15 isolates (five from grapes and 10 from winery), showed unique biotype.

## Discussion

Nowadays, the issue of the *B. bruxellensis* origins in the winemaking field remains under debate and has never been clearly demonstrated. The origins of these spoilage yeast in the vineyard has been long neglected in favor of the winery (Ibeas et al., 1996). This has probably been due to their poor detection on grapes, because of their low growth and competition ability, poor surface adhesion, the use of chemical treatments, possible entrance in a VBNC state and possible negative interactions with other microorganisms (Arvik and Henick-Kling, 2002; Schifferdecker et al., 2014; Coton et al., 2017). Indeed, isolation of *B. bruxellensis* from a complex yeast community generally requires differential medium formulations that include cycloheximide, to eliminate the sensitive fastest growing species (Rodrigues et al., 2001; Morneau et al., 2011). However, *B. bruxellensis* was probably not isolated from grapes due to the lack of specific cultivation methods and due their presence in very low numbers. The combined use of the specific enrichment liquid medium (i.e., EBB) and the selective DBDM agar medium (the *Dekkera/ Brettanomyces* differential medium) (Rodrigues et al., 2001; Renouf and Lonvaud-Funel, 2007; Garijo et al., 2015) has now allowed the isolation of *B. bruxellensis* strains from grapes.

The present study has confirmed the previous failure to directly isolate *B. bruxellensis* from the grape surface, through our use of the EBB selective medium. Thus, we isolated *B. bruxellensis* from eight samples out of 26 using this combination of enrichment and selective media. In contrast, during the must fermentation, *B. bruxellensis* was directly detected with quantitative levels from 10^2^ CFU/mL to 10^5^ CFU/mL, in seven of the total of 21 samples. Indeed, although *B. bruxellensis* isolates are usually present as a minority species in the winery environment, they can increase in number during the more nutritionally advantageous conditions, while exploiting their slow metabolism (Fugelsang et al., 1993). This can occur in particular once the alcoholic fermentation is almost complete, when the trace levels of residual sugars could allow *B. bruxellensis* to proliferate (Oelofse et al., 2008). Indeed, wine will represent a suitable ecological niche for some *B. bruxellensis* strains that can exploit its chemical characteristics, such as the presence of oxygen (i.e., pumping over; a wooden barrel), the sufficient nitrogen levels, the ethanol presence, the low pH, and the presence of various vitamins (Smith and Divol, 2016).

However, in the present study in the winery environment, the spread of *B. bruxellensis* was limited. Indeed, no *B. bruxellensis* were detected in the air, or in the trailer or the destemmer. This agrees with previous studies (Donnelly, 1977), although it is in contrast with (Stratford, 2006), who reported *Brettanomyces/Dekkera* in the air samples taken near to barrels located in an area that communicated with the outdoors. Other studies have reported the presence of *B. bruxellensis* in wineries using a combination of culture-based methods and direct detection with molecular probes, and particularly for equipments that are difficult to sanitize, such as cement storage vessels and oak barrels (Fugelsang, 1997; Curtin et al., 2015). In agreement with their results, we isolated *B. bruxellensis* from the valves, where must and wine can remain during the transfer operations from one fermenter to another. Although these connections are washed, some *B. bruxellensis* cells might survive, to find favorable conditions for their colonization and multiplication, which creates a dangerous and uncontrolled transit system in the winery.

Regarding the uncertain origins of *B. bruxellensis* wine strains, in the present study some aspects of the ecology of these yeasts were elaborated suggesting the grape surfaces as primary sources. The clustering of the biotypes did not show any clear separation between the vineyard and winery strains, but rather the presence of the same biotypes on the grapes and in the winery. Indeed, analyzing all *B. bruxellensis* molecular profiles after characterization procedure, it can be noted that biotypes include several isolates coming from vineyard and winery, without a clear distinction between the two environments. In this specific condition (the large distance 50 Km, the exclusion of any human flux from winery to vineyard) the grape surface could represents the primary source of these *B. bruxellensis* even if other contamination ways such as insects or birds could be not theoretically excluded. In this regard, to support this hypothesis further investigations involving more years and/or other vineyards and wineries needed, also in the light of the patterns depicted studying the diversity of the model organism *S. cerevisiae* on a comparable scale (Knight and Goddard, 2015). On the other hand, the wine and some of the winery equipments are more suitable ecological niche for *B. bruxellensis* colonization, growth and diffusion. Indeed, almost all of *B. bruxellensis* strains that were isolated from the grapes showed the same biotypes as those recovered in the winery (i.e., from the valves and the final stages of grape must fermentation). Furthermore, the absence of *B. bruxellensis* strains from the “Merlot” grapevine variety from the same environment indicates that the grape variety can influence the presence of certain yeast, in agreement with Raspor et al. (2006) and Cordero-Bueso et al. (2011), who reported strong correlations among grape varieties and yeast biota.

In conclusion, the results of the present study show that *B. bruxellensis* can be isolated (with great difficulty) from grapes. We did not find distinct populations between strains from grapes and strains from winery, but the presence of the same biotypes in both environments was observed. In the condition tested, this statement suggests a *B. bruxellensis* flux from the grapes to the winery even if more investigations (subsequent vintages and/or more vineyards and wineries) are necessary to generalize this evidence. *B. bruxellensis* reveals a great metabolic plasticity, as its appear to quickly adapt to the winery environmental. The differences between grape varieties might play a role for the presence and diffusion *B. bruxellensis* in the winemaking environment, although further investigations are needed to confirm this hypothesis.

## Author Contributions

LO, LC, VM, MC, and FC contributed equally to this manuscript. All authors participated in the design and discussion of the research. LO and VM carried out the experimental part of the work. LC, LO, MC, and FC carried out the analysis of the data and wrote the manuscript. All authors have read and approved the final manuscript.

## Conflict of Interest Statement

The authors declare that the research was conducted in the absence of any commercial or financial relationships that could be construed as a potential conflict of interest.
