# Supplementary figures and images for: Occurrence of Brettanomyces bruxellensis on Grape Berries and in Related Winemaking Cellar
Source: Front Microbiol. 2019 Mar 7;10:415. doi: 10.3389/fmicb.2019.00415 (PMC6416197; doi:10.3389/fmicb.2019.00415)

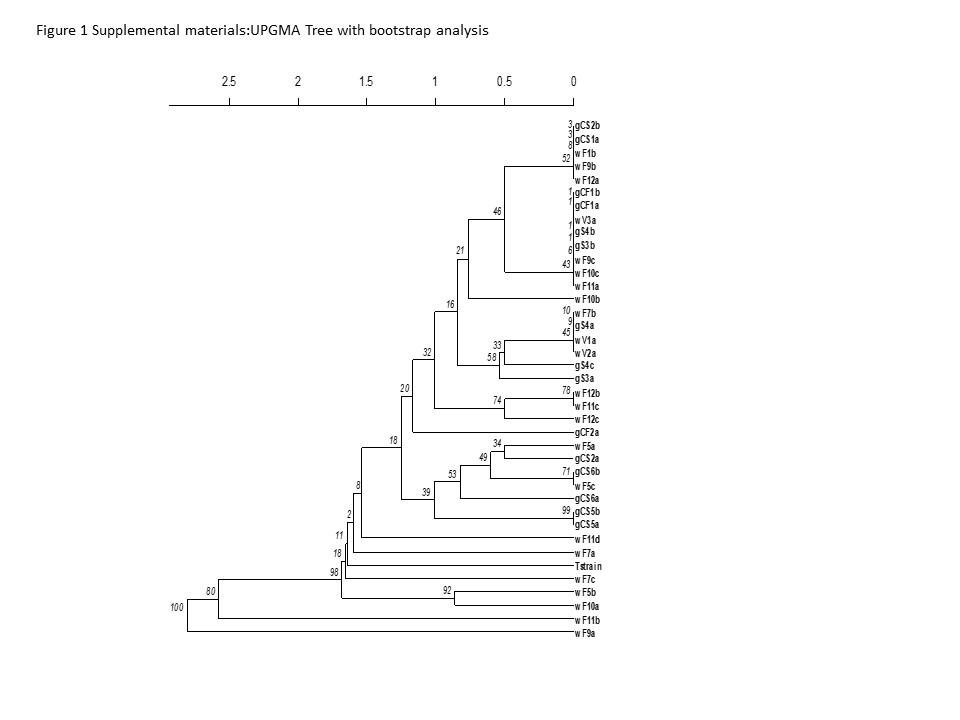

Supplement: Supplementary file 1 [file Image_1.JPEG]
